# Impact of a mass media campaign on bed net use in Cameroon

**DOI:** 10.1186/1475-2875-12-36

**Published:** 2013-01-25

**Authors:** Hannah L Bowen

**Affiliations:** 1 Impact Programs, Malaria No More, 432 Park Ave S, New York, NY, 10016, USA

**Keywords:** Malaria, Communication, Mass media, Behaviour change, BCC, ITN, LLIN, Mosquito net, Bed net, Net use

## Abstract

**Background:**

In 2011, Cameroon and its health partners distributed over eight million free long-lasting insecticide treated nets (LLINs) in an effort to reduce the significant morbidity and mortality burden of malaria in the country. A national communications campaign was launched in July 2011 to ensure that as the nets were delivered, they would be used consistently to close a net use gap: only 51.6% of adults and 63.4% of their children in households with at least one net were sleeping under nets before the distribution. Even in households with at least one net for every two people, over 35% of adults were not sleeping under a net. Malaria No More (MNM) adapted its signature NightWatch communications programme to fit within the coordinated “KO Palu” (Knock Out Malaria) national campaign. This study evaluates the impact of KO Palu NightWatch activities (that is, the subset of KO Palu-branded communications that were funded by MNM’s NightWatch program) on bed net use.

**Methods:**

Using national survey data collected at baseline (in March/April 2011, before the national LLIN distribution and KO Palu NightWatch launch) and post-intervention (March/April 2012), this study evaluates the impact of exposure to KO Palu NightWatch activities on last-night net use by Cameroonian adults and their children under five. First, a plausible case for causality was established by comparing net use in 2011 and 2012 and measuring exposure to KO Palu NightWatch; next, a propensity score matching (PSM) model was used to estimate the impact of exposure on net use by simulating a randomized control trial; finally, the model was tested for sensitivity to unmeasured factors.

**Results:**

The PSM model estimated that among Cameroonians with at least one net in their household, exposure to KO Palu NightWatch activities was associated with a 6.6 percentage point increase in last-night net use among respondents (65.7% *vs* 59.1%, p < 0.05) and a 12.0 percentage point increase in last-night net use among respondents’ children under five (79.6% *vs* 67.6%, p < 0.025). Sensitivity analysis suggests only a very small risk of bias from omitted factors influencing exposure and net use.

**Conclusions:**

Extrapolating the results of the PSM model to the population of Cameroonians with access to at least one mosquito net, this analysis estimates that approximately 298,000 adults and over 221,000 of their children under five slept under a bed net because of the knowledge, motivation, and/or timely reminder provided by KO Palu NightWatch activities. The programme cost less than $0.16 per adult reached, and less than $1.62 per additional person protected by a net. The results suggest a strong role for mass media communication interventions in support of investments in malaria control commodities such as LLINs.

## Background

According to the World Health Organization (WHO)’s World Malaria Report 2011, every one of Cameroon’s 19.6 million citizens is at risk of malaria, with 71% of them living in high transmission areas [1]. Over 1.8 million suspected cases of malaria were recorded countrywide in 2010, along with over 4,500 recorded malaria-attributed deaths. Malaria was estimated to be responsible for 19% of under-five child deaths in 2008, more than any other single cause [2].

In 2011, Cameroon made great strides in malaria control by making life-saving commodities available across the country. With funding from the Global Fund to Fight HIV/AIDS, Tuberculosis, and Malaria and support from WHO and the Roll Back Malaria partnership (RBM), the government and its health partners procured and distributed over eight million free, long-lasting insecticide treated nets (LLINs) to prevent malaria in Cameroon’s first ever universal coverage net distribution. Other malaria control activities included a pilot programme to use rapid diagnostic tests (RDTs) in health facilities and through community health workers that was brought to national scale in the second half of 2012; and subsidized first-line treatment with artemisinin-based combination therapy (ACT). The effectiveness of these interventions has been studied extensively, and the current analysis therefore treats bed net use as a proxy for final health outcomes (reduction in malaria cases and deaths) based on the findings of bed net efficacy studies [3,4]. In order for these life-saving commodities to actually save lives, however, they must be used appropriately and consistently. Previous studies have investigated the barriers to use and reasons for non-use of bed nets, identifying such factors as knowledge about the causes of malaria, type and condition of nets, socio-economic indicators including education, and perceptions of heat/discomfort under a net; a study in Zambia suggested that behaviour change communication “are necessary to improve use” and could “contribute towards closing the gap between ownership and use” [5-7].

This article focuses on the impact of a mass media campaign on the use of one critical commodity: bed nets. One of the key challenges in implementing a universal coverage net distribution is ensuring that the LLINs (received for free by eligible households regardless of their prior interest in obtaining or using a mosquito net) are used consistently. Throughout this article, last-night net use is used as a proxy for consistent use, as the most reliable indicator that can be captured during a one-time interview. A baseline survey of malaria knowledge, attitudes and practices (KAP) conducted by MNM in March/April 2011 (unpublished survey conducted prior to the net distribution), found that Cameroon’s “net use culture” was moderate; 51.6% of adults in households with at least one mosquito net slept under a net the night before the survey, and 63.4% said their children had used a net. Even in households with enough nets to cover all sleeping spaces (self-reported level of coverage), there was a “usage gap” of 30.5% of adults and 24.5% of their children who did not sleep under the available net the night before.^a^ Results from the 2011 DHS/MICS survey of Cameroon (using a somewhat different methodology, counting only confirmed insecticide treated nets (ITN) and LLINs, and conducted from September to November 2011) found only 43.4% of children under five in households with at least one ITN slept under an ITN the previous night and 34.5% of individuals (all ages) slept under an ITN [8].

To strengthen Cameroon’s net use culture and bolster the use of newly distributed LLINs in particular (as opposed to the previously available untreated nets or ITNs that needed frequent retreatment), a coalition of health partners, including the Ministry of Health, National Malaria Control Program (NMCP), United Nations Children’s Fund (UNICEF), Plan International, Institut pour la Recherche le Développement Socio-économique et la Communication (IRESCO), Clinton Health Access Initiative (CHAI), Cameroon Coalition Against Malaria (CCAM), Association Camerounaise pour le Marketing Social (ACMS-PSI), MNM, and private sector partners COTCO, ExxonMobil, and MTN implemented a national malaria communication campaign under a unified brand, the “KO Palu” (Knock Out Malaria) campaign. Within the KO Palu campaign, MNM took the lead on mass media activities, adapting its signature communications platform, NightWatch, to the Cameroonian context (NightWatch has also been implemented in Senegal, Chad, and Tanzania). The subset of KO Palu activities carried out by MNM are referred to throughout this article as “KO Palu NightWatch.”

The NightWatch platform is grounded in theories of behaviour change and social mobilization that describe how communication feeds into a process of individuals and communities recognizing the importance of a public health threat, learning about methods of prevention and/or treatment, taking action, and thereby improving health outcomes [9-11]. The core conceptual model for NightWatch hypothesizes that communication contributes to malaria-control behaviours, such as net use, in two ways: supporting individual action (by imparting knowledge, influencing attitudes, and providing consistent and motivational reminders) and shaping social norms. Key evidence-based NightWatch strategies include using credible and popular local celebrities as messengers, distributing consistent messages across multiple mass media outlets, and reinforcing messages over time [12-14].

One year after measuring baseline KAP indicators, and eight months after launching KO Palu NightWatch activities, MNM conducted an impact evaluation using a post-exposure cross-sectional survey. The results were intended to both measure the impact of the mass media campaign on bed-net usage and identify opportunities to adapt the campaign’s strategy for future work in Cameroon.

## Methods

### Programme activities and theory of causality

**Figure 1:**
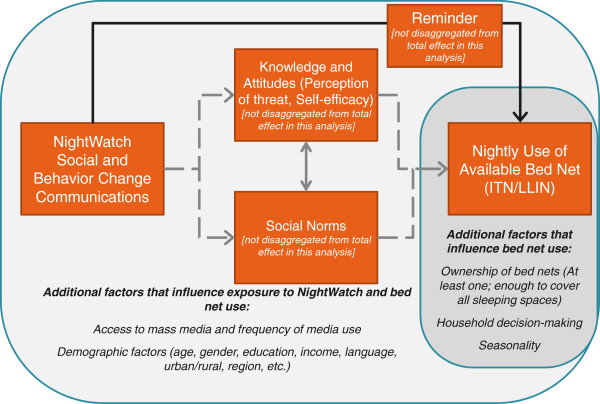
**NightWatch theory of causality.** Hypothesized channels through which NightWatch directly and indirectly impacts malaria control behaviour.

The challenges of attributing causality to mass media campaigns’ impact on public health outcomes are well established, arising from the inherent lack of a comparable control group as well as the difficulty of disaggregating the role of mass media campaigns from the other public health interventions they are typically designed to complement. Kincaid and Do suggest that post-intervention, cross-sectional population surveys can provide reliable estimates of impact that are generalizable to the population, so long as threats to internal validity are addressed during the analysis stage [15]. As the authors point out, however, to be persuasive the analysis must also be grounded in a plausible theory of causality. MNM’s KO Palu NightWatch activities were designed based on a hypothesis of both direct and indirect impacts on net-use behaviour (see Figure 1). This section describes the KO Palu NightWatch activities and their intended impact on behaviour.

With significant programme support from ExxonMobil and COTCO, MNM implemented a number of mass media activities under the KO Palu NightWatch programme between July 2011 and March 2012. These included:

### KO Palu anthem

The anthem is an original song and music video featuring educational lyrics about malaria control, performed by popular Cameroonian artists Petit Pays, Sine, Mercellin Ottou, Frederique Ottou, Lady Ponce and Richard Bona. The anthem was released at a NightWatch press conference in July 2011 and featured at the August 2011 KO Palu Campaign launch event. Distribution went viral immediately, with the song becoming popular across the whole spectrum of local radio and the music video appearing on local television, regional satellite stations like *Trace TV*, and even the diaspora-facing channel *Afrotainment*. The music video was also viewed by over 33,000 people on *YouTube* (as of September, 2012). CDs began popping up for sale on street corners (without being produced by MNM) and the anthem was heard throughout Yaoundé’s popular bars and clubs.

### KO Palu NightWatch PSAs

Four PSAs were released in August 2011 and aired on *CRTV, Equinox TV, Canal 2 International, and Spectrum TV*, as well as 16 radio stations. The PSAs featured singer Lady Ponce delivering a message in French and NBA player Luc Mbah a Moute in English. The first wave of PSAs was on-air daily at 9 pm from 29 July to 29 October, 2011. Three additional TV PSAs (with messages from Sine in English, Princess Khadizah in Fulfulde, and XMaleya in French) and seven additional radio PSAs (with messages from Akon in English, Afo Akom in Pidgin, Frederique Ottou in French, Princess Khadizah in Fulfulde, Sine in English and French, XMaleya in English and French, and a combination message in French and English from XMaleya, Sine, Pit Baccardi, and Afo Akom) were on air at least once daily from 1 April to 1 July, 2012.^b^

#### KO Palu NightWatch SMS

In addition to donating media distribution of the PSAs, MTN made an in-kind contribution of net-use reminders via SMS to MTN subscribers. A first round of SMS reminders began in August 2011, and a second round began in February/March 2012; the SMS featured one of three key messages in either English or French.

### Other activities

Widely publicized events included the NightWatch press conference in July 2011, KO Palu campaign launch in Yaoundé in August 2011, and press conferences making critical announcements regarding the timing and logistics of net distribution activities, which had been delayed. MTN also sponsored approximately 100 billboards featuring KO Palu celebrity spokespeople, posted around high-traffic areas throughout Cameroon in early 2012. The KO Palu Campaign has also produced hand-out materials such as 2012 calendars and sponsored events in Yaoundé and Douala to celebrate World Malaria Day in April 2012.

MNM’s 2011–2012 KO Palu NightWatch activities were designed to complement the large-scale, free LLIN distribution, the aim of which was to distribute approximately one net for every two household members. The potential impact of the mass media campaign can only be understood within the context of rapidly expanded access to nets. In particular, MNM and other health partners were interested in the effectiveness of the mass media campaign as a behaviour-change tool to ensure that nets, when available, are used consistently (as captured in the surveys by last-night net use). Naturally, receipt of free LLINs, even in the absence of any communication programme, would likely have a large impact on the percentage of the total population using nets. Similarly, an increase in the number of nets per household could have impacted household allocation of nets. The goal of this impact evaluation is to determine the additional impact of the KO Palu NightWatch activities on net use behaviour over and above greater access to nets. Therefore, the analysis includes only respondents with at least one mosquito net at home, and controls for ownership of more nets.

During the study period (March 2011 to March 2012), the KO Palu NightWatch activities were the primary mass media communications about malaria in Cameroon. Other health partners such as IRESCO also released KO Palu-branded communications in early 2012, but they focused on case management (diagnosis and treatment) as opposed to net use. This analysis focuses only on the KO Palu NightWatch activities conducted by MNM, and may underestimate the full impact of the KO Palu brand if those considered “not exposed” did actually benefit from other partners’ communication activities under the unified brand.

### Study design

The programme evaluation described here is based on a post-intervention cross-sectional survey (the “2012 Malaria KAP Survey”). In addition to the cross-sectional data, initial evidence of a plausible relationship (that is, that audience members recalled KO Palu NightWatch communications and that behaviour did change after the communications were distributed) is based on year-on-year changes from baseline pre-intervention cross-sectional data (the “Malaria KAP Baseline”). The Malaria KAP Baseline questionnaire was developed by MNM in 2011, drawing on key malaria indicators used by RBM’s Monitoring and Evaluation Reference Group (MERG), standard Malaria Indicator Survey (MIS) and Demographic and Health Survey (DHS) questionnaires, and consultation with health partners in Cameroon. The 2012 Malaria KAP Survey questionnaire used the Baseline questions, supplemented with additional questions on exposure to KO Palu NightWatch activities and participation in the 2011 national universal coverage net distribution (registration and receipt of free LLINs). The questionnaire was pretested in English, French, and Pidgin on March 17–18, 2012 prior to fieldwork.

The Malaria KAP Baseline consisted of face-to-face pen-and-paper interviews with 2,566 randomly selected respondents (see “Sample and data processing” for details of random selection in both 2011 and 2012). The 2012 Malaria KAP Survey consisted of 2,176 interviews. Fieldwork for both surveys was conducted by TNS-RMS, an internationally recognized market research firm with a permanent office in Douala, Cameroon. Fieldwork was carried out in March/April both years, removing any risk that trends over time are due to seasonal variation in net use. TNS-RMS adhered to strict ethical guidelines, including obtaining informed consent from all respondents prior to conducting interviews and registering the study with the Comité National d’Ethique du Cameroun in March 2011. All TNS-RMS interviewers underwent extensive training prior to conducting fieldwork, including an overview of the study background and purpose; methodology and sampling, including household and respondent selection; proper use of survey materials; confidentiality and survey ethics; and a detailed review of questionnaire administration, question-by-question.

### Sample and data processing

The sampling frame consisted of the full population of adults over age 15 in Cameroon’s 10 regions, estimated at 10,940,736 [16]. A representative sample was selected using multi-stage random sampling (proportionate to population size). First, interviews were allocated according to the urban and rural population of each region, given in the 2005 national census (detailed breakdowns of the 2010 Census were not available at the time of sampling), and then distributed to sampling points in each region as follows: (1) urban locations were selected purposively, including all regional capitals except in the south-west region, where Buea was replaced by Limbe due to its greater weight in the region’s population and level of development; Douala and Yaoundé were considered as cosmopolitan urban areas; (2) rural areas were randomly selected among villages/towns located within approximately 150 km of the selected urban areas; sector selection was made in 2011 and the same sectors used in 2012 except for five sampling points substituted due to limited accessibility; (3) each selected urban centre and rural sector was broken down into a mutually exclusive but exhaustive sampling grid on a map, and sampling points randomly selected (resampled each year) among the sampling grids with a maximum 10 interviews per sampling point, according to the allocation of interviews to each sector in the previous step; (4) at each sampling point, households were selected by a random walk methodology; (5) at each selected household, one respondent was selected using a Kish Grid [17]. The final sample had demographic characteristics similar to recent national statistics (see Table 1).

**Table 1:** Demographic characteristics of survey sample

	**National Institute of Statistics**	**2011 Malaria KAP Baseline**	**2012 Malaria KAP Survey**
**Location**			
Urban	52%*	51%	50%
Rural	48%*	49%	50%
**Gender**			
Male	49%*	49%	50%
Female	51%*	51%	50%
**Age (% of adults 15+ in each range)**			
15-29	51%*	50%	54%
30+	49%*	46%	42%
No answer		4%	4%
**Education**			
No formal schooling, no response	17%**	13%	7%
Primary	33%**	20%	23%
Secondary (1st cycle)	28%**	30%	32%
Secondary (2nd cycle)	15%**	24%	26%
Higher/University	7%**	13%	12%
**Religion**			
Islam	20%**	22%	25%
Traditional religion	3%**	1%	1%
Catholicism	37%**	38%	38%
Christianity (others)	35%**	33%	34%
Other, no response	4%**	5%	3%
**Household goods**			
Mobile Phone	--	77%	82%
Television	--	78%	80%
Radio	--	76%	74%
VCD or DVD player	--	65%	66%
Refrigerator	--	30%	32%

* Data source: République du Cameroun, Institut National de la Statistique. La Population du Cameroun en 2010. [http://www.statistics-cameroon.org/downloads/La_population_du_Cameroun_2010.pdf].

** Data source: Institut National de la Statistique, Ministère de l’Économie, de la Planification et de l’Aménagement du Territoire, Ministère de la Santé Publique, MEASURE DHS ICF International: Enquête Démographique et de Santé et à Indicateurs Multiples EDS-MICS CAMEROUN 2011 Rapport Préliminaire. Yaoundé; 2011.

Completed paper questionnaires were transported from all regions to TNS-RMS’ main data processing centre in Douala, Cameroon for coding and data entry. Data were entered by experienced data entry clerks using double entry and strict quality control, cleaned, and delivered to MNM in SPSS format. After comparing key demographic statistics of the achieved sample with the 2011 Malaria KAP Baseline and 2010 Census data, it was decided that no corrective weighting of the data would be necessary.

### Definition of key variables

Since the KO Palu NightWatch activities were designed to focus primarily on encouraging consistent use of bed nets by all Cameroonians, the key outcome for measuring programme impact is a proxy for consistent net use: last-night net use by children and adults. The analysis considers only households with at least one net; the impact being evaluated is thus restricted to individuals’ and households’ decisions to sleep under a net when one is available. The role of the KO Palu NightWatch activities in stimulating demand for the free LLINs is not captured in this analysis.

### Outcome variables

The two outcomes of interest are self-reported last-night net use by respondents, and last-night net use by respondents’ children under five, as reported by parents/caregivers and not differentiated by number of children (one or more children under five slept under a net the previous night *vs* no children under five slept under a net the previous night).

### Campaign exposure variables

Campaign exposure is measured by a composite of eight individual measures of campaign activity recall: two radio PSAs (French and English, heard on the radio at least once), two television PSAs (French and English, saw on television at least once), bed net reminder SMS (ever received; only included if the respondent said the SMS came from MTN), KO Palu Anthem (heard audio at least once), KO Palu Anthem (saw video at least once), KO Palu Launch Event (heard about, saw, or attended). Once the composite variable was created, it was split at the median (recall of two or more campaign activities). Of the respondents who had at least one bed net in their households, 50.3% were “exposed” to KO Palu NightWatch by this definition, with another 11.4% recalling only one activity and 38.3% recalling none. Activities by other health partners under the unified KO Palu brand were not included in the composite.

### Covariates

A number of demographic characteristics and other factors potentially influence exposure to KO Palu NightWatch activities, consistent use of bed nets, or both. The factors included in this analysis were: gender, age (measured in years), education (highest level attained, measured as a scale from 0–2), religion, presence of children in the household, language (understanding of French), urban/rural location, region (two of three zones with similar socio-economic characteristics, malaria burdens, and baseline net-use behaviour: zone 1 includes Northwest and Southwest; zone 2 includes Littoral, Adamawa, North and Far North), media access (household ownership of television, radio, or mobile phone), media use (daily television viewing), socio-economic status (two scales indicating numbers of basic goods and luxury goods owned by the household), registration for the universal coverage net distribution (self-reported), bed net ownership (self-reported ownership of enough bed nets to cover all sleeping spaces in the household), and knowledge about bed nets (spontaneous mention of bed nets as a method of preventing malaria).

### Analysis

Analysis was conducted in Stata version 10 on a merged dataset of the 2011 and 2012 data files. Initially, year-on-year comparisons using independent sample t-tests (unequal variances assumed) were conducted to determine whether net use behaviour had changed during the period that the KO Palu NightWatch activities were hypothesized to be impacting behaviour. Once a behaviour change coinciding with the mass media campaign was identified, subsequent analysis included only the 2012 data. Logistic regression models for each behavioural outcome were constructed to estimate the marginal impact of exposure to KO Palu NightWatch on behaviour, controlling for observed covariates.

Though the logistic regression models account for many possible confounders, there remains a risk of selection bias. Those exposed to the mass media campaign and those not exposed likely differ in ways that also influence behavioural outcomes; those not exposed do not offer a valid counterfactual for what would have been the behaviour of the exposed group without the KO Palu NightWatch campaign. The next step in the analysis, therefore, was to use a propensity score-matching (PSM) model to simulate a valid control group.

Babalola and Kincaid (2009) suggest that PSM can provide an estimate of the impact of health communications that is internally valid; additional sensitivity testing and analysis by biprobit regression can be used to corroborate the results and test for the influence of unmeasured covariates [18]. PSM is widely recognized as a powerful technique for estimating average treatment effects by simulating a randomized control design using post-intervention, cross-sectional survey data [19-21]. It is a technique that lends itself well to mass media interventions, where a control is not possible when implementing programmes at full scale, but has been used in only a limited number of studies, and rarely for malaria communications.

## Results

### Descriptive

The 2012 Malaria KAP survey found significant increases in access to and use of bed nets in Cameroon, a testament to the coordinated efforts of government and health partners in malaria control. Table 2 presents progress from 2011 to 2012 in the primary outcome variables, as well as exposure to KO Palu NightWatch activities during the study period. Net use increased dramatically at the national level from 30.6% to 52.3% (p < 0.000) of all adults and from 41.1% to 65.4% (p < 0.000) among their children under five. At the same time, over 60% of respondents recalled at least one of the KO Palu activities funded by MNM’s NightWatch programme.

**Table 2:** Key indicators, 2011 and 2012

	**2011**		**2012**		**Increase year-on-year**	**p-value**
	**n**	**%**	**n**	**%**	**Percentage Points**	**(Difference in Means, Independent Samples T-Test)**
**All respondents**	**2566**	**100%**	**2176**	**100%**		
≥1 net in HH	1467	57%	1791	82%	25.14	0.000
Enough nets in HH to cover all sleeping spaces	652	25%	1,291	59%	33.92	0.000
Slept under a net the previous night	785	31%	1,138	52%	21.71	0.000
**Respondents in HH with ≥1 net**	**1467**	**100%**	**1791**	**100%**		
Slept under a net the previous night	757	52%	1,121	63%	10.99	0.000
**Respondents in HH with enough nets for all sleeping spaces**	**652**	**100%**	**1,291**	**100%**		
Slept under a net the previous night	453	69%	901	70%	0.31	0.888
**All parents/caregivers of children under five**	681	100%	833	100%		
Child(ren) slept under a net the previous night	280	41%	545	65%	24.31	0.000
**Parents/caregivers in HH with ≥1 net**	**426**	**100%**	**730**	**100%**		
Child(ren) slept under a net the previous night	270	63%	540	74%	10.59	0.000
**Parents/caregivers in HH with enough nets for all sleeping spaces**	**188**	**100%**	**540**	**100%**		
Child(ren) slept under a net the previous night	142	76%	415	77%	1.32	0.717
**All respondents**	**-**		**2176**	**100%**		
Recalled ≥1 KO Palu NightWatch Activity	-		1,318	61%		
Recalled ≥2 KO Palu NightWatch Activity	-		1,061	49%		
Recalled KO Palu Anthem	-		1,000	46%		
Recalled KO Palu Anthem music video	-		983	45%		
Recalled KO Palu PSA on TV	-		570	26%		
Recalled KO Palu PSA on Radio	-		232	11%		
Recalled KO Palu bed net reminder SMS from MTN	-		483	22%		
Recalled KO Palu launch event	-		220	10%		

Progress on net use indicators from 2011 to 2012, as well as reach of KO Palu NightWatch activities during the study period.

**Figure 2:**
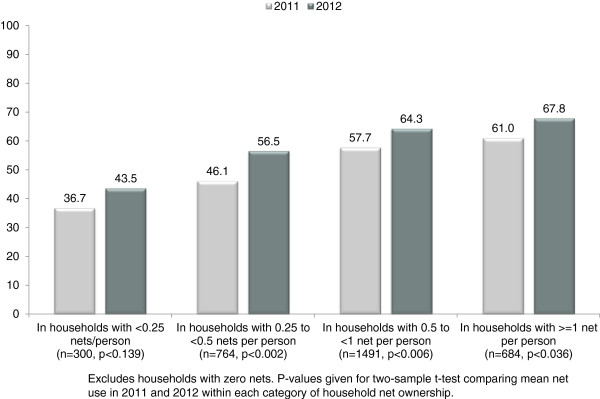
**Adults’ last night net use, by household’s level of access to bed nets.** Year on year comparison of net use among households with different levels of bed net coverage for their household members.

As the year-on-year comparison shows, net use increased not only in a broad sense (largely thanks to the success of the net distribution), but also among respondents with at least one net in their household. Figure 2 breaks this group down further, and shows that net use increased among respondents living in households with low access to nets (at least one net, but fewer than 0.25 nets per household member), with greater but still limited access (between 0.25 and 0.5 nets per household member), and with so-called “universal access” (0.5 nets per household member or more). This suggests that access was not the only factor determining net use, and that a stronger net use culture may indeed be developing. Further, these changes in net use year-on-year cannot be explained by seasonal variation in net use. Though net use may be higher during the rainy season, both surveys were conducted at the end of the dry season, in March/April.

One prerequisite for suggesting a causal link between the mass media campaign and net use is satisfied by observing an increase in net use not fully explained by increased access to nets at either the national or household level. The other is a high enough level of exposure to the campaign to allow for comparison. The KO Palu NightWatch mass media activities were nearly ubiquitous in Cameroon, though some target populations such as rural residents were less likely to recall the campaign activities. The KO Palu Anthem, which was played on both television and radio consistently throughout the study period, was the most widely recognized element of the campaign. The lower reach of specific television and radio PSAs is likely due more to the stepwise fashion they were rolled out at two points in the study period (with some time off-air) than to their resonance with the audience, though this will be investigated in 2013 when PSAs have been broadcast consistently for a longer period of time. Though 30.3% of all respondents recalled receiving “an SMS that reminded you to sleep under a bed net,” the analysis includes only those who spontaneously mentioned MTN as the source of the message (a restriction that lowers the exposure measure from 30.3% to 22.2%). The composite variable based on recall of all MNM-funded KO Palu NightWatch activities defined 50.3% of respondents as “exposed.”

The hypothesis being tested is that mass media campaign exposure helps explain the increase in net use by respondents with at least one net at home (11 percentage points from 51.6% to 62.6% for adults’ own use, p < 0.000, and 10.6 percentage points from 63.4% to 74.0% for children’s use, p < 0.000). Since the study was not a panel design, the remainder of the analysis looks only at 2012 Malaria KAP Survey data to isolate explanatory factors. Without controlling for any other factors, individuals with at least one net at home who were exposed to KO Palu NightWatch (by the definition of recalling at least two elements of the campaign) were 7.3 percentage points (66.2% *vs* 58.9%, p < 0.001) more likely to have slept under a bed net the previous night than those with at least one net at home who were not exposed, and 12.1 percentage points (79.9% *vs* 67.8%, p < 0.000) more likely to have had their children sleep under a net the previous night. At first glance, this suggests that net use increased across the board over 2011 levels, but increased more for those who recalled KO Palu NightWatch than for those who did not. The analyses that follow look at the extent to which that difference among 2012 Malaria KAP Survey respondents is actually attributable to KO Palu NightWatch itself, rather than other confounding factors.

### Logistic regression

**Figure 3:**
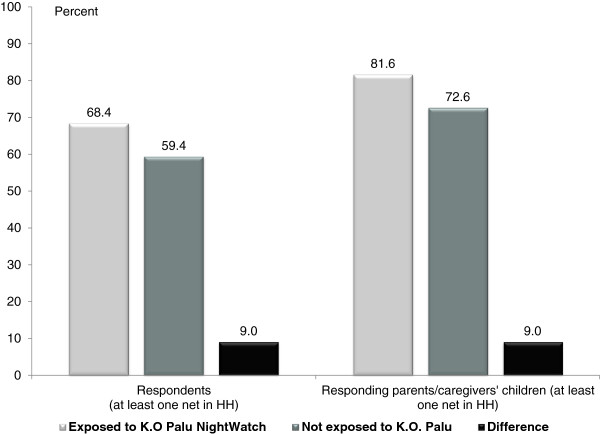
**Marginal effects of KO Palu NightWatch on net use.** Marginal effects of KO Palu NightWatch on last-night net use: results of logistic regression model with covariates held at mean values.

The first estimate of the impact of KO Palu NightWatch activities on bed net use consists of a logistic regression model that controls for other determinants of net use, but not for possible endogeneity of the exposure variable. Tables 3 and 4 present the odds ratios generated by each model and demonstrate that mass media campaign exposure is a strong and statistically significant predictor of net use for both adults and their children (odds ratio of 1.482, p < 0.001 for adults, and odds ratio of 1.677, p < 0.009 for their children). Calculating the marginal effects of campaign exposure for individuals with at least one net at home with all other variables constant at mean values, an individual exposed to the campaign was 9 percentage points more likely to sleep under a net (68% *vs* 59%) and 9 percentage points more likely to put their child(ren) under a net (82% *vs* 73%) (see Figure 3). According to this estimate, confounding factors such as education, location, and net ownership have different influences on the estimate of adults’ *vs* children’s net use. While this may be the case if the underlying factors shaping adults’ decision-making differ for their own net use and their children’s, it could also indicate that the logistic regression models do not sufficiently control for the underlying factors shaping net use behaviour so that the marginal effects are biased estimators of impact.

**Table 3:** Multivariate logistic regression, dependent variable: respondent used a net “last night”

	**Odds ratio**	**Robust standard error**	**P-value**	**95% confidence interval**	
**Exposure to KO Palu NightWatch**					
Recall zero/one element	1.000				
Recall two or more elements of campaign	1.482	0.174	0.001	1.177	1.864
**Age**					
Each additional year, 15-64	1.013	0.005	0.015	1.002	1.023
**Gender**					
Male	1.000				
Female	1.292	0.143	0.021	1.040	1.604
**Education**					
No formal education	1.000				
Each additional level: primary, more than primary	1.309	0.144	0.014	1.055	1.624
**Religion**					
Islam or other	1.000				
Christianity	1.464	0.193	0.004	1.130	1.897
**Children**					
None	1.000				
At least one in household	1.279	0.140	0.025	1.032	1.585
**Location**					
Urban	1.000				
Rural	0.782	0.092	0.037	0.621	0.985
**Region**					
Centre, East, West, South, North-west, South-west	1.000				
Adamawa, Far North, Littoral, North	0.445	0.054	0.000	0.351	0.564
**Socio-economic Status**					
Do not own basic goods (television, radio, mobile phone, or DVD/VCD player)	1.000				
Number of basic household goods owned	0.695	0.038	0.000	0.624	0.774
**Net ownership**					
At least one net in household	1.000				
Enough nets for all sleeping spaces in household	3.164	0.377	0.000	2.505	3.997
**Knowledge of bed nets**					
Did not mention bed nets for malaria prevention	1.000				
Mentioned bed nets for malaria prevention	1.455	0.197	0.006	1.116	1.898

N = 1,717 respondents with at least one bed net in household.

Likelihood ratio χ^2^(11) = 218.52.

P-value = 0.0000.

Pseudo R^2^ = 0.1168.

**Table 4:** Multivariate logistic regression, dependent variable: respondents’ child(ren) used a net “last night”

	**Odds ratio**	**Robust standard error**	**P-value**	**95% confidence interval**	
**Exposure to KO Palu NightWatch**					
Recall zero/one element	1.000				
Recall two or more elements of campaign	1.677	0.330	0.009	1.141	2.466
**Religion**					
Islam or other	1.000				
Christianity	1.569	0.314	0.024	1.060	2.321
**Location**					
Urban	1.000				
Rural	0.456	0.090	0.000	0.310	0.672
**Region**					
Centre, East, West, South	1.000				
Adamawa, Far North, Littoral, North	0.278	0.067	0.000	0.174	0.446
North-west, South-west	0.552	0.170	0.054	0.302	1.010
**Socio-economic Status**					
Do not own basic goods (television, radio, mobile phone, or DVD/VCD player)	1.000				
Number of basic household goods owned	0.783	0.065	0.003	0.666	0.921
**Net ownership**					
At least one net in household	1.000				
Enough nets for all sleeping spaces in household	1.709	0.351	0.009	1.144	2.555
**Knowledge of bed nets**					
Did not mention bed nets for malaria prevention	1.000				
Mentioned be.d nets for malaria prevention	1.643	0.398	0.041	1.022	2.641

N = 730 responding parents/caregivers with at least one bed net in household.

Likelihood ratio χ^2^(8) = 88.07.

P-value = 0.000.

Pseudo R^2^ = 0.1190.

### Propensity score-matching

**Figure 4:**
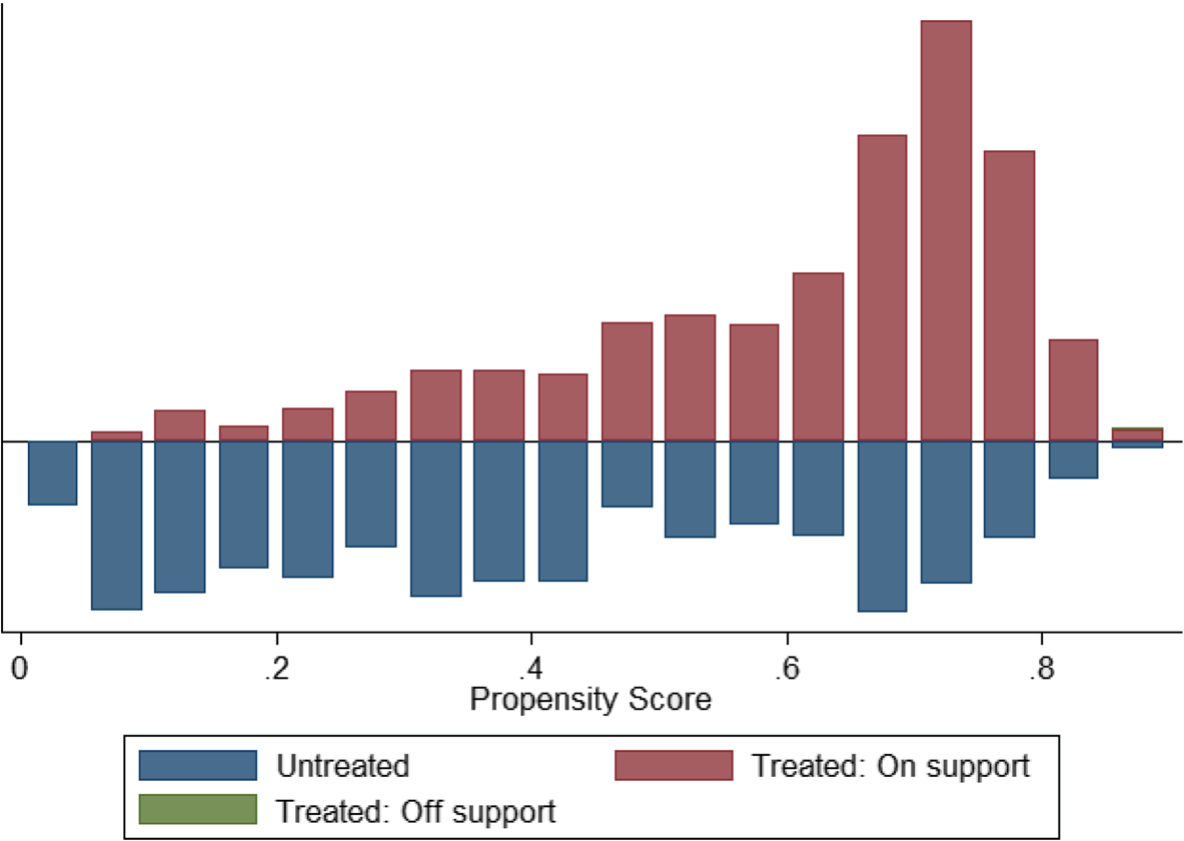
**Propensity scores (adults’ net use PSM model).** Distribution of propensity scores among treatment and control group, model of net use by adults.

**Figure 5:**
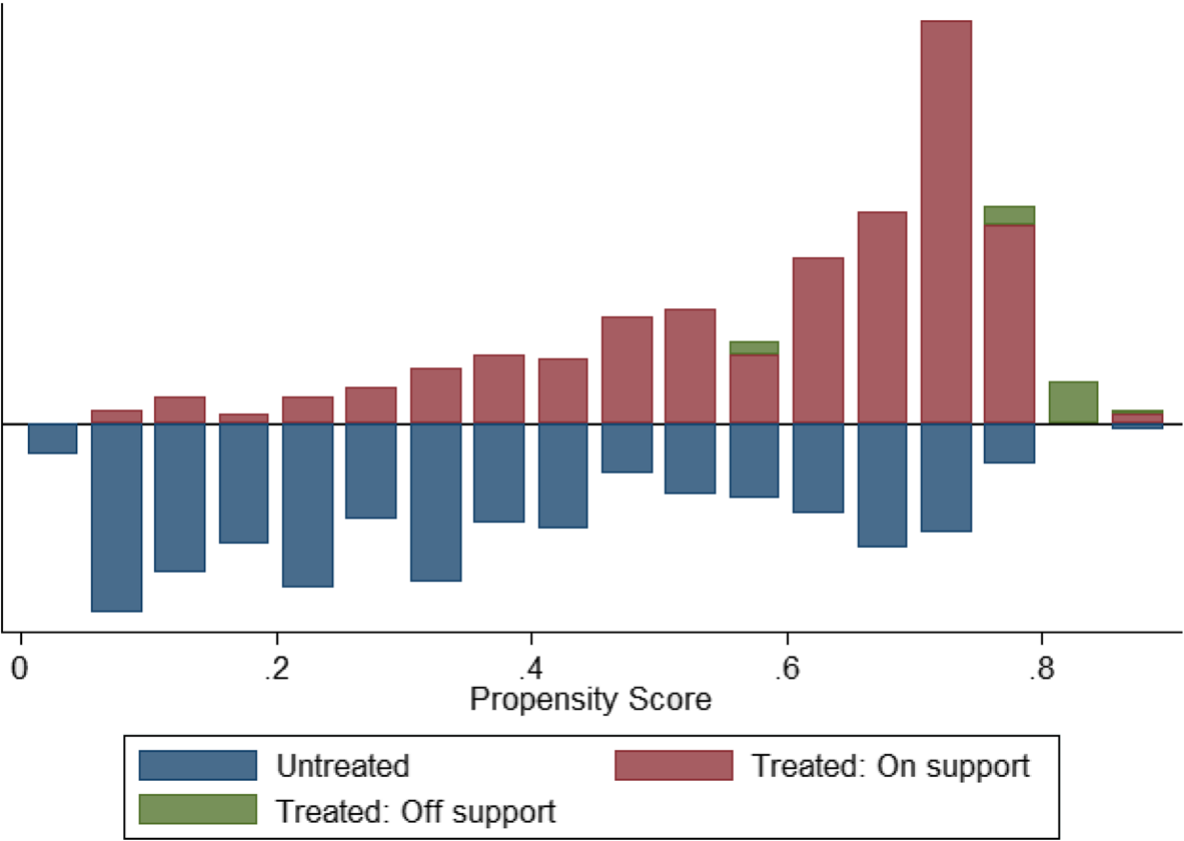
**Propensity scores (children’s net use PSM model).** Distribution of propensity scores among treatment and control group, model of net use by respondents’ children.

As noted above, the estimates from simple logistic regression models are subject to potential bias from inherent differences between those exposed to KO Palu NightWatch activities and those not exposed. Therefore, a new model was estimated using propensity scores based on the measured covariates to simulate a valid comparison group and then calculate the average treatment effect on the treated (ATT, the impact of exposure to the mass media campaign on net use among those exposed). The propensity score represents an individual’s likelihood of being exposed to KO Palu NightWatch, regardless of whether they were actually exposed or not. Scores were calculated using Stata command *pscore* and predictor variables for gender, age, education, urban/rural location, region (one of three zones), frequency of television viewing, radio ownership, ownership of luxury goods, and participation in the universal coverage free net distribution [22]. Scores were calculated from 1,717 respondents with at least one net at home, and ranged from 0.0183 to 0.8938 with mean 0.5104 (see Figures 4 and 5 for distributions of propensity scores in the adults’ and children’s net use models). The procedure generated eight subgroups in which all key variables were balanced (that is, independent of exposure to KO Palu NightWatch), except for one: one of the binary variables indicating region (North-west, South-west) was not balanced in the first subgroup. The resulting matched treatment (exposed) and control (not exposed) groups did not differ significantly on most key demographic variables (see Tables 5 and 6). One of the dangers of including a large number of matching variables is that it becomes increasingly difficult to balance all characteristics of the treatment and control group. However, the remaining differences in French language capacity and television ownership (for adults) are not concerning given the balanced regional distribution (which approximates accessibility to target audiences better than language) and daily television use (which is more directly relevant than television ownership). The remaining differences in gender (for children’s net use) are unlikely to bias the results since respondents’ gender was not related to children’s net use. For children’s net use, the remaining differences in regional distribution and mobile phone ownership are more concerning, but since the matched controls were somewhat wealthier on average than the matched treatment group it may be safe to assume that any potential bias resulting from region or mobile phone use may be mitigated by household socio-economic status.

**Table 5:** Group characteristics before and after matching: adult net use

	**Without matching**			**After matching**		
	**Exposed**	**Not exposed**	**p > |t|**	**Matched treated**	**Matched control**	**p > |t|**
Gender (binary, 1 = female)	0.49	0.53	0.113	0.49	0.49	0.886
Age (years)	28.41	31.77	0.000	28.41	28.17	0.635
Have child(ren) in household (binary)	0.55	0.53	0.417	0.55	0.54	0.533
Understand French (binary)	0.83	0.65	0.000	0.84	0.79	0.009
Understand English (binary)	0.19	0.19	0.841	0.19	0.18	0.422
Education (scale, 0-3)	1.84	1.45	0.000	1.85	1.86	0.485
Urban/rural (binary, 1 = rural)	0.40	0.62	0.000	0.40	0.41	0.662
Region (binary, 1 = Adamawa, Far North, Littoral, North)	0.46	0.49	0.116	0.46	0.47	0.632
Region (binary, 1 = North-west, South-west)	0.12	0.23	0.000	0.12	0.13	0.350
Watch television daily (binary)	0.86	0.52	0.000	0.86	0.86	0.836
Own a television (binary)	0.93	0.68	0.000	0.93	0.87	0.000
Own a radio (binary)	0.84	0.69	0.000	0.84	0.85	0.646
Own a mobile phone (binary)	0.89	0.76	0.000	0.89	0.86	0.051
Own luxury goods (scale for number of goods)	1.87	1.03	0.000	1.87	1.83	0.738
Number of nets per household member (ratio)	0.73	0.67	0.002	0.73	0.66	0.001

Mean characteristics of respondents with at least one net in their household, before and after matching.

**Table 6:** Group characteristics before and after matching: children’s net use

	**Without matching**			**After matching**		
	**Exposed**	**Not exposed**	**p > |t|**	**Matched treated**	**Matched control**	**p > |t|**
Gender (binary, 1 = female)	0.64	0.65	0.689	0.65	0.72	0.049
Age (years)	29.66	32.13	0.001	29.69	29.51	0.796
Understand French (binary)	0.85	0.65	0.000	0.85	0.85	0.830
Understand English (binary)	0.15	0.18	0.355	0.15	0.19	0.190
Education (scale, 0-3)	1.82	1.36	0.000	1.82	1.82	0.926
Urban/rural (binary, 1 = rural)	0.43	0.66	0.000	0.43	0.47	0.399
Region (binary, 1 = Adamawa, Far North, Littoral, North)	0.48	0.52	0.237	0.48	0.57	0.027
Region (binary, 1 = North-west, South-west)	0.11	0.25	0.000	0.12	0.10	0.541
Watch television daily (binary)	0.87	0.49	0.000	0.87	0.84	0.387
Own a television (binary)	0.94	0.66	0.000	0.94	0.91	0.157
Own a radio (binary)	0.82	0.67	0.000	0.82	0.86	0.143
Own a mobile phone (binary)	0.90	0.75	0.000	0.90	0.85	0.040
Own luxury goods (scale for number of goods)	1.79	0.98	0.000	1.63	1.90	0.049
Number of nets per household member (ratio)	0.64	0.58	0.005	0.63	0.62	0.654

Mean characteristics of respondents with at least one net in their household, before and after matching.

**Figure 6:**
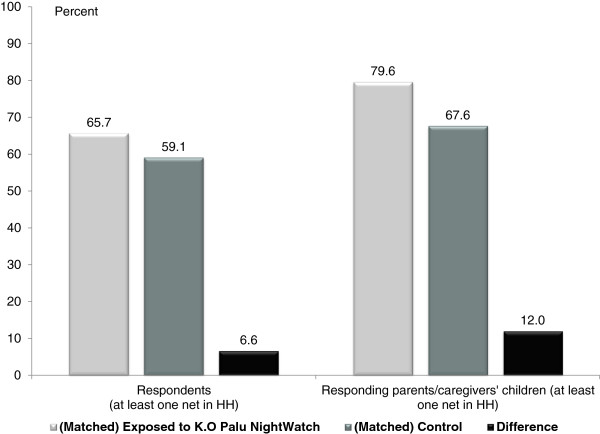
**Impact of KO Palu NightWatch on net use.** Results of propensity score-matching model of the impact of KO Palu NightWatch on last-night net use.

Calculating ATT using Stata command *psmatch2* for nearest-neighbour matching with a .005 caliper and the propensity scores generated by *pscore* yielded estimates of a 6.6 percentage point impact on respondents’ net use (p < 0.05) and a 12.0 percentage point impact on their children’s net use (p < 0.025) [23]. Figure 6 illustrates the difference in bed net usage between those who recalled the KO Palu NightWatch activities and the matched control group.

The estimates of KO Palu NightWatch exposure on adult net use have the same direction and significance as the logistic regression models. The PSM estimations of ATT, however, are closer in magnitude to the unmatched estimates and the correction for bias is more logical, moving in the same direction for both adults’ and children’s net use. The correction is larger for adults’ net use, which stands to reason if adult net use is more sensitive to other factors. For example, the data from both 2011 and 2012 suggest that when households do not have enough nets to cover all sleeping spaces, they tend to cover children first and leave adults unprotected.

### Sensitivity analysis and bivariate probit

A first test of the robustness of the results was to re-run the PSM analysis on only households with enough nets for all sleeping spaces, to check whether the impact of KO Palu NightWatch exposure held up even in the case of full access. The balancing property for the PSM model is not satisfied as fully for this specification, and the group (individuals with enough nets in their household to cover all sleeping spaces) is less generalizable; still, the findings confirm the direction and significance of the initial model. In fact, the analysis on this subgroup yields an estimate of impact (ATT) on adult net use of 8.5 percentage points (72.9% *vs* 64.3%, p < 0.01) and on children’s net use of 19.3 percentage points (84.0% *vs* 64.8%, p < 0.005).

Three additional methods were used to test for possible unmeasured confounders and are described briefly: sensitivity analysis using the *sensatt* command in Stata, examination of the potential influence of unobserved confounders using the *mhbounds* command in Stata, and testing for evidence of such unobserved confounders using a bivariate probit model [18].

Using *sensatt* to re-estimate the impact (ATT) of KO Palu NightWatch (with a PSM model that includes a simulated confounder) suggests that the impact is insensitive to the omission of plausible unobserved confounders [24]. Two tests were run: one with a confounder that is positively associated with both KO Palu NightWatch exposure and last-night net use (the omission of which could result in overestimates of impact), and one with a confounder that is negatively correlated with exposure but positively correlated with net use (the omission of which could result in underestimates of impact). As shown in Table 7, neither simulation changes the estimate of ATT.

**Table 7:** Sensitivity analysis

	**Estimate of ATT**	**Standard error**	**Outcome effect (Odds ratio)**	**Selection effect (Odds ratio)**
**Last-night net use (respondents)**				
Original PSM Model (using *psmatch2*)	0.066	0.037		
Original PSM Model (using *sensatt*)	0.072	0.036		
PSM Model with simulated confounder #1	0.072	0.036	1.100	0.863
PSM Model with simulated confounder #2	0.072	0.036	2.770	1.236
**Last-night net use (responding parents/caregivers’ children)**				
Original PSM Model (using *psmatch2*)	0.120	0.058		
Original PSM Model (using *sensatt*)	0.110	0.056		
PSM Model with simulated confounder #1	0.110	0.056	1.339	0.956
PSM Model with simulated confounder #2	0.110	0.056	3.199	1.398

Estimates of ATT with and without simulated confounder.

Using the Mantel-Haenszel bounds test as described by Becker and Caliendo to gauge the potential impact of positive or negative bias of various sizes on the estimate of impact, the author finds reason to be more cautious [25]. The impact estimates could lose their statistical significance when an unobserved confounder is assumed to cause overestimation of impact; based on the estimated bounds, the treatment effect on last-night net use by adults would not be statistically significant once such an omitted variable increased the odds by 15% (p_mh+ > 0.1 for Γ = 1.15) that someone who would use a net anyway would be exposed to KO Palu NightWatch. The treatment effect on last-night net use by respondents’ children is somewhat more robust, but would lose statistical significance with an omitted variable that increased the odds of KO Palu NightWatch exposure by 45% (p_mh+ > 0.1 for Γ = 1.45). While this result suggests some sensitivity to potential unobserved bias, it seems unlikely that there is another variable, even something that cannot be measured (such as motivation), that would have so large an impact on exposure and net use. The concern raised by the M-H bounds estimate is also somewhat alleviated by the *sensatt* analysis, which included variables with a strong positive selection effect, simulating a factor as important as gender, without invalidating the estimate of impact.

Finally, a bivariate probit regression model can detect evidence of omitted confounding variables by identifying any correlation between the residual terms of a system of equations that simultaneously describe both KO Palu NightWatch exposure and last-night net use. If there were omitted variables that influence both exposure and net use, the correlation of residuals (rho) would be significantly different from zero. Table 8 summarizes biprobit models that show a positive, but not statistically significant, impact of KO Palu NightWatch exposure on net use. The estimates are less precise than the PSM model, with Model 1 estimating marginal effects of exposure to be 13.7 percentage points and Model 2 estimating marginal effects of exposure to be 11.1 percentage points. Neither model, however, shows evidence of any omitted confounders that jointly influence exposure and net use. For Model 1 (adult net use), the correlation between residuals (rho) is not significantly different from zero (−0.068, p > 0.642); similarly, the null hypothesis (no correlation between residuals) cannot be rejected in Model 2 (children’s net use, 0.001, p > 0.995).

**Table 8:** Biprobit analysis for omitted variables that influence exposure and net use

	**Model 1: Adult net use**			**Model 2: Children's net use**		
	**Coefficient**	**Standard Error**	**P-value**	**Coefficient**	**Standard Error**	**P-value**
N = respondents with at least one bed net in household	n = 1717			n = 696		
**Last-night net use**						
KO Palu NightWatch exposure: Recall two or more activities	0.360	0.233	0.122	0.361	0.292	0.217
Age: each additional year, 15-64	0.008	0.003	0.013	-	-	-
Gender: female	0.162	0.066	0.014	-	-	-
Education: each additional level: primary, more than primary	0.150	0.083	0.072	-	-	-
Religion: Christianity	0.244	0.082	0.003	0.251	0.128	0.050
At least one child in household	0.144	0.066	0.028	-	-	-
Location: rural	-	-	-	-0.467	0.126	0.000
Region: Adamawa, Far North, Littoral, North	-0.479	0.073	0.000	-0.607	0.123	0.000
Socio-economic status: number of basic household goods owned	-0.209	0.035	0.000	-0.157	0.056	0.005
Net accessibility: enough nets for all sleeping spaces in household	0.695	0.072	0.000	0.382	0.120	0.001
Knowledge: mentioned bed nets for malaria prevention	0.222	0.082	0.007	0.295	0.141	0.036
Constant	-0.471	0.197	0.017	0.860	0.274	0.002
**Exposure to KO Palu NightWatch activities**						
Age: each additional year, 15-64	-0.010	0.003	0.001	-0.014	0.006	0.022
Gender: female	-0.064	0.066	0.333	-0.207	0.120	0.085
Education: each additional level: primary, more than primary	0.465	0.070	0.000	0.506	0.107	0.000
Location: rural	-0.117	0.073	0.107	-0.181	0.113	0.109
Region: Adamawa, Far North, Littoral, North	-0.141	0.074	0.056	-0.347	0.123	0.005
Region: North-west, South-west	-0.558	0.097	0.000	-0.740	0.159	0.000
Media use: watch television daily	0.737	0.080	0.000	0.871	0.133	0.000
Media access: household owns a radio	0.239	0.083	0.004	0.260	0.130	0.046
Socio-economic status: number of luxury goods owned	0.058	0.020	0.004	0.050	0.035	0.147
Registered for universal coverage net distribution	0.199	0.113	0.078	0.134	0.224	0.552
Constant	-1.177	0.222	0.000	-0.826	0.401	0.039
**Correlation between residuals**	**-0.068**	**0.146**	**0.641**	**0.001**	**0.193**	**0.995**

Altogether, the results of sensitivity analysis using *sensatt*, M-H bounds, and a biprobit regression model suggest that the assumptions underlying the PSM model are valid, and not sensitive to bias from plausible unobserved variables. The point estimates from PSM vary somewhat based on the specification and method of estimation, but remain positive, significant, and of similar magnitude.

### Cost analysis

Taking the estimates of ATT given by the PSM model, it is possible to estimate the cost of the KO Palu NightWatch communications by two measures: per individual reached, and per additional individual sleeping under a mosquito net. Program costs of $842,966.34 cover the period January 2011-March 2012, and include MNM staff support (75% of all staff costs in Cameroon, as well as pro-rated staff costs for New York support staff), in-country and international travel for MNM staff, Cameroon country office overhead, production of campaign materials, media distribution (counted at actual discounted rates, rather than full market value), and monitoring and evaluation. The cost estimate for reach is a conservative estimate since it only includes adults who recalled the KO Palu NightWatch activities; it is likely that many children would also see or hear what their parents are watching on television or listening to on the radio. In addition, 14% of respondents who were exposed to KO Palu NightWatch activities said that they frequently discuss malaria communications with friends or family members, creating an unmeasured indirect audience. Even with this conservative estimate, however, the programme cost only $0.13 to $0.16 per adult reached (see Table 9).

**Table 9:** KO Palu NightWatch cost estimates

**Total population (2010 estimate)**	**19,406,100**	
**Total programme cost (January 2011-March 2012)**	**$842,966.34**	
	Projection of survey results to full population	Cost per person reached/ protected
**Reach (upper estimate):** adults who recalled at least one KO Palu NightWatch activity	6,626,788	$0.13
**Reach (lower estimate):** adults who recalled at least two KO. Palu NightWatch activities	5,334,614	$0.16
**Impact (upper estimate):** marginal increase in net use attributable to KO Palu NightWatch exposure, applied to population of adults and children under five in households with at least one net (assume 61.6% exposure to at least one KO Palu NightWatch activity)	637,835	$1.32
**Impact (lower estimate):** marginal increase in net use attributable to KO Palu NightWatch exposure, applied to population of adults and children under five in households with at least one net (assume 50.3% exposure to at least two KO Palu NightWatch activities)	519,974	$1.62

Estimates of cost per person reached and per person protected by a net as a result of exposure to KO Palu NightWatch.

The cost per person protected by a net because of KO Palu NightWatch is higher, since some of those exposed to KO Palu NightWatch would have slept under a net even without the reminder. This cost estimate is also conservative, assuming only one child under five per responding parent/caregiver (since the increase in net use was estimated per adult respondent’s household, rather than per child), and leaving out the behaviour of five to 14 year olds, whose behaviour was not analysed in this study. Still, the cost per person protected by a net because of KO Palu is quite low, between $1.32 and $1.62 (see Table 9). To put these costs in context, increasing net use by providing more nets would be six to seven times more costly assuming each LLIN cost approximately $10 to deliver to a household in Cameroon (cost of the net itself and distribution).

## Discussion

### Campaign impact

Evaluating the impact of a mass media campaign is notoriously difficult due to the lack of a valid comparison group. However, using propensity score-matching to construct equivalent groups of individuals exposed to the campaign and not exposed to the campaign, it is possible to estimate the average treatment effect. The ATT describes the impact of exposure to KO Palu NightWatch communications on net use among those exposed to the messages, controlling for other factors. The analysis included only respondents who had at least one mosquito net in their household, since the impact under investigation is the consistent use of available nets. Based on the PSM model, adults exposed to KO Palu NightWatch communications were 6.6 percentage points more likely to sleep under a mosquito night the previous night (65.7% *vs* 59.1%) and 12.0 percentage points more likely to have their child(ren) sleep under a net (79.6% *vs* 67.6%). Extrapolating to the population 15 years or older with at least one net at home, this represents a conservative estimate of 298,650 adults and over 221,000 of their children under five that slept under a bed net because of the knowledge, motivation, and/or timely reminder provided by KO Palu NightWatch activities.^c^

### Practical and policy implications

These findings suggest an important role for mass media behaviour change communications in ensuring that available bed nets are used appropriately to prevent malaria. For malaria control programs seeking to ensure that their investments in bed nets do not go to waste, the findings offer a possible route to close the gap between net access and net use.

Though it has proven difficult to attribute causality to mass media interventions for malaria control behaviour in the past, this study uses post-intervention, cross-sectional survey data and propensity score-matching to simulate a randomized control study. Given the plausible theory of causality, positive and significant impact estimates (even after controlling for other explanatory factors and testing for sensitivity of the model) provide strong evidence of the role of mass media communications in encouraging net use. Further studies will be necessary to test whether the impact is similar in other environments, and whether the impact persists over time. In addition, the malaria community would benefit from studies that are better able to disaggregate the impact of various mass media channels (including radio, television, and SMS) in specific contexts. Finally, MNM and other partners intend to use mass media campaigns to support appropriate use of diagnostic and treatment commodities (RDTs and ACT) as well as nets in the coming years and, therefore, need to evaluate whether mass media communications are as effective on treatment-seeking behaviour as on net use behaviour.

### Limitations

One of the main challenges of assessing the full impact of KO Palu NightWatch activities on bed net use was the lack of precise measures of social norms and beliefs that might have been influenced by the campaign and, in turn, encouraged net use. This is in fact one of the indirect channels through with NightWatch programs are intended to work, but is virtually impossible to track through quantitative research.

Another limitation is the potential to generalize from these results. Though the survey sample was broadly representative of the Cameroonian population in comparison to recent Census and DHS survey data, somewhat higher levels of education in the MNM survey samples suggest that some of the most vulnerable populations may have been slightly undersampled. In addition, countries with different levels of access to LLINs or different starting net use cultures could respond differently to a NightWatch programme.

## Conclusions

National surveys of Cameroon in 2011 and 2012 found evidence of significant increases in last-night use of mosquito nets among adults and children under five, even when comparing usage only among individuals with at least one net at home. Using the 2012 dataset to identify drivers of this increased net use, this analysis shows a significant impact from Malaria No More’s KO Palu NightWatch activities, even after controlling for other factors. A propensity score-matching model using individuals with at least one net at home, which simulated a control group against which to compare the behaviour of individuals exposed to KO Palu NightWatch, found that exposure increased adults’ net use by 6.6 percentage points (from 59.1% among controls to 65.7% among those exposed) and increased their children’s net use by 12.0 percentage points (from 67.6% among controls to 79.6% among those exposed). This translates into over 500,000 individuals that used a mosquito net to protect themselves from malaria as a result of the KO Palu NightWatch, at a cost of less than $1.62 per person protected.

## Endnotes

^a^In addition to measures of the number of nets and number of household members, the practical level of coverage was assessed using a survey question, “Do you have mosquito nets for all sleeping spaces in your household; including beds as well as any spaces that are used as sleeping spaces at night?” In both counting nets and assessing the coverage of sleeping spaces, the survey included all nets without distinguishing ITNs and LLINs.

^b^Messages in the PSAs included:

Protégez-vous et votre famille contre le paludisme. N'oubliez pas de dormir sous vos moustiquaires imprégnées ce soir.

Did you know that the mosquito that transmits malaria primarily bites at night? A treated mosquito net is one of the best ways to protect you and your family from malaria.

Saviez-vous que vous ne pouvez obtenir le paludisme qu’à partir d’une piqûre de moustique? Une moustiquaire imprégnée est l'une des meilleures façons de vous protéger, vous et votre famille contre le paludisme.

Did you know that anyone can get malaria no matter what age, so everyone needs to protect themselves and sleep under a mosquito net.

Chaque enfant mérite le meilleur début dans la vie, ils ont des rêves, ils ont des espoirs ils ont des aspirations. Donnez à vos enfants une chance. Protégez-les contre le paludisme. Le paludisme est une maladie préventive et soignable. Assurez-vous que vos enfants et vous dormiez sous une moustiquaire imprégnée chaque nuit pour que leurs rêves se réalisent.

^c^Calculation based on total population of 19,406,100; 10,940,736 adults 15 years and older; 9,004,990 adults with at least one net in their household; and 3,670,376 adults with at least one net and at least one child under five. Impact calculated as the difference between predicted number of net users using actual KO Palu exposure among those with at least one net (assume 50.3% of those with at least one net at home slept under a net at the rate predicted by the PSM model for “exposed to KO Palu NightWatch” and 49.7% of the population slept under a net at the rate predicted by the PSM model for controls) and predicted number of net users if all adults with at least one net at home slept under a net at the rate predicted by the PSM model for controls.

## Competing interests

The author declares that she has no competing interests.

## Authors’ contributions

HB led the design of the study, carried out the statistical analysis, and drafted the manuscript.

## References

[B1] WHO World Malaria Report 2011 2011 Geneva http://www.who.int/malaria/world_malaria_report_2011/en/

[B2] WHO Cameroon: Health statistics profile 2010 2010 African Health Observatory http://www.who.int/gho/countries/cmr/en

[B3] Lengeler C Insecticide-treated bed nets and curtains for preventing malaria Cochrane Database Syst Rev 2004 2 CD000363 15106149 10.1002/14651858.CD000363.pub2

[B4] Eisele TP Larsen DA Walker N Cibulskis RE Yukich JO Zikusooka CM Steketee RW Estimates of child deaths prevented from malaria prevention scale-up in Africa 2001–2010 Malar J 2012 11 93 10.1186/1475-2875-11-93 22455864 PMC3350413

[B5] Macintyre K Littrell M Keating J Hamainza B Miller J Eisele TP Determinants of hanging and use of ITNs in the context of near universal coverage in Zambia Health Policy Plan 2012 27 316 325 10.1093/heapol/czr042 21652576

[B6] Baume CA Franca-Koh AC Predictors of mosquito net use in Ghana Malar J 2011 10 265 10.1186/1475-2875-10-265 21920034 PMC3196744

[B7] Ndo C Menze-Djantio B Antonio-Nkondjio C Awareness, attitudes and prevention of malaria in the cities of Douala and Yaoundé (Cameroon) Parasit Vectors 2011 4 181 10.1186/1756-3305-4-181 21933411 PMC3192766

[B8] Institut National de la Statistique, Ministère de l’Économie, de la Planification et de l’Aménagement du Territoire, Ministère de la Santé Publique, MEASURE DHS ICF International Enquête Démographique et de Santé et à Indicateurs Multiples EDS-MICS CAMEROUN 2011 Rapport Préliminaire 2011 Yaoundé http://www.statistics-cameroon.org/downloads/EDS-MICS11/EDS_MICS_2011_Rapport_preliminaire_27_oct_11.pdf

[B9] Rosenstock IM Strecher VJ Becker MH Social learning theory and the health belief model Health Educ Q 1988 15 175 183 10.1177/109019818801500203 3378902

[B10] Bandura A Social Foundations of Thought and Action: A Social Cognitive Theory 1986 Englewood Cliffs, N.J.: Prentice-Hall

[B11] Hornik RC Public Health Communication: Evidence for Behavior Change 2002 Mahwah, New Jersey: LEA

[B12] Horton D Wohl RR Mass communication and parasocial interaction: Observations on intimacy at a distance Psychiatry 1956 19 215 229 13359569 10.1080/00332747.1956.11023049

[B13] Brown WJ Basil MD Bocarnea MC The influence of famous athletes on health beliefs and practices: Mark McGwire, child abuse prevention, and androstenedione J Health Commun 2003 8 41 57 10.1080/10810730305733 12635810

[B14] Gerbner G Gross L Morgan M Signorielli N The “mainstreaming” of America: violence profile No. 11 J Commun 1980 30 10 29

[B15] Kincaid DL Do MP Multivariate causal attribution and cost-effectiveness of a national mass media campaign in the Philippines J Health Commun 2006 11 Suppl 2 69 90 17148100 10.1080/10810730600974522

[B16] UN Statistics Division Population by age, sex and urban/rural residence, 2010 Updated 31 Jan 2012 [http://data.un.org/Data.aspx?q=cameroon+population+by+age&d=POP&f=tableCode:22;countryCode:120&c=2,3,5,7,9,11,13,14,15&s=_countryEnglishNameOrderBy:asc,refYear:desc,areaCode:asc&v=1]

[B17] Gaziano C Comparative analysis of within-household respondent selection techniques Public Opin Q 2005 69 124 157 10.1093/poq/nfi006

[B18] Babalola S Kincaid DL New methods for estimating the impact of health communication programs Commun Methods Meas 2009 3 61 83 10.1080/19312450902809706

[B19] Dehejia RH Wahba S Propensity score matching methods for non-experimental causal studies Rev Econ Stat 2002 84 151 161 10.1162/003465302317331982

[B20] Heckman JJ Ichimura H Todd P Matching as an econometric evaluation estimator Rev Econ Stat 1998 65 261 294

[B21] Caliendo M Kopeinig S Some practical guidance for the implementation of propensity score matching J Econ Surv 2008 22 31 72 10.1111/j.1467-6419.2007.00527.x

[B22] Becker SO Ichino A Estimation of average treatment effects based on propensity scores Stata J 2002 2 358 377

[B23] Leuven E Sianesi B PSMATCH2: Stata module to perform full Mahalanobis and propensity score matching, common support graphing, and covariate imbalance testing Statistical Software Components, Boston College Department of Economics 2003 http://ideas.repec.org/c/boc/bocode/s432001.html

[B24] Nannicini T Simulation-based sensitivity analysis for matching estimators Stata J 2007 7 334 350

[B25] Becker SO Caliendo M Sensitivity analysis for average treatment effects Stata J 2007 7 71 83

